# Effect of histamine strength and devices on skin prick test (SPT) response following antihistamine inhibition

**DOI:** 10.1186/1710-1492-8-S1-A3

**Published:** 2012-11-02

**Authors:** Greg Plunkett, Robert Erskine, Joshua Young

**Affiliations:** 1 LK-Abello, Inc, Round Rock, Texas, 78664 USA

## Background

A histamine positive control (HPC) is used in skin prick testing in order to make sure that the patient has a valid wheal/erythema response. Antihistamines and other factors can suppress the skin response to allergens. The purpose of this study is to evaluate different histamine concentrations and SPT devices with antihistamine suppressed subjects.

## Methods

SPT was performed on subjects using multiple allergen extracts and 6mg/mL and 1mg/mL histamine base with devices from 2 manufacturers; Lincoln Diagnostics and Hollister-Stier. Some subjects were tested with diluted Timothy grass extract. A single dose of antihistamine, cetirizine, was taken and SPT performed for up to 72hrs.

## Results

Suppression of wheal responses was significant following antihistamine up to 20 hr for both HPC and most allergens (15% to 70% wheal size in mm). 1 mg/mL histamine appeared negative in some cases under suppression when the 6mg/mL histamine was positive. The lowered wheal size persisted in some subjects up to 72 hrs. Even though the HPC did not appear to be suppressed at some times following antihistamine due to the cutoffs chosen, some allergens that were positive before antihistamines were clearly suppressed and scored a negative result even with positive HPC. SPT results showing suppression also depended on the device. The Lincoln device had a 1.5-3 mm larger wheal than the Hollister-Stier device.

## Conclusions

Some allergens remained suppressed even when the HPC and other allergens returned to their original wheal size. This suggests that antihistamine suppression is not equal across allergens and may result in false negative diagnoses.

